# Cover Crop Management on the Southern High Plains: Impacts on Crop Productivity and Soil Water Depletion

**DOI:** 10.3390/ani11010212

**Published:** 2021-01-16

**Authors:** Lisa L. Baxter, Charles P. West, C. Philip Brown, Paul E. Green

**Affiliations:** 1 Department of Crop and Soil Sciences, University of Georgia, Tifton, GA 31793, USA; 2 Department of Plant and Soil Science, Texas Tech University, Lubbock, TX 79409, USA; chuck.west@ttu.edu (C.P.W.); philip.brown@ttu.edu (C.P.B.); paul.green@ttu.edu (P.E.G.)

**Keywords:** Southern High Plains, Ogallala Aquifer, cover crop, annual forage, teff, semi-arid

## Abstract

**Simple Summary:**

Agriculture throughout the Southern High Plains of the United States relies on supplemental irrigation from the Ogallala Aquifer because of limited precipitation and high evapotranspiration rates. Unfortunately, extraction rates greatly exceed the recharge rate of the aquifer. This region is prone to soil erosion, so maintaining soil coverage is critical for protecting soil resources. Despite the many benefits of planting cover crops, producers do not readily plant winter crops in the Southern High Plains due to concerns of depleting soil water availability for subsequent summer crops. Based on the results of this experiment, producers can plant rye with a no-till drill to help maintain soil cover and produce high-quality winter forage for grazing livestock without threatening soil water supply for the summer crops; in this case, teff. Light irrigation (up to 25 mm per month) should be applied to supplement rainfall and ensure grazing if the producer is reliant upon the rye and does not have other forage available for contingency. Finally, timely termination of the cover crops is critical. Delayed termination of the cover crops was the biggest factor found to reduce the productivity of the teff.

**Abstract:**

The imminent depletion of the Ogallala Aquifer demands innovative cropping alternatives. Even though the benefits of cover crops are well recognized, adoption has been slow in the Southern High Plains (SHP) of the United States because of concerns that cover crops withdraw soil water to the detriment of the summer crops. This small plot experiment tested the interacting effects—production, soil water depletion of the cover crops, and subsequent teff [*Eragrostis tef* (Zucc.) Trotter] summer hay crops—of irrigation and tillage management with five cover crop types to identify low-risk cover crop practices in the drought-prone SHP. Dryland rye (*Secale cereale* L.) produced modest forage biomass (>1000 kg ha^−1^), even in a dry year, but it was found that light irrigation should be used to ensure adequate forage supply (>1200 kg ha^−1^) if winter grazing is desired. No-till management and timely termination of the winter cover crops were crucial to reducing the negative impact of winter crops on summer teff production. The results indicated no detriment to soil water content that was attributable to planting no-till cover crops compared with the conventional practice of winter fallow. Therefore, producers could take advantage of the soil-conserving attributes of high-quality winter forage cover crops without experiencing significant soil water depletion.

## 1. Introduction

Agriculture on the Southern High Plains (SHP) relies on supplemental irrigation from the Ogallala Aquifer, which stretches across eight states and underlies more than 440,000 sq. km [1]. Unfortunately, the longevity of the Ogallala Aquifer is threatened because the extraction rates of approximately 300 mm per year in the South Plains of Texas greatly exceed the annual estimated recharge rate of 13 mm [1]. With strong gusts of wind and limited precipitation (470 mm is the annual mean), the soil is prone to severe erosion; this is especially true during the winter, when most annual croplands are fallow [2]. The use of cover crops as forage can supplement producers’ income to mitigate losses resulting from reduced pumping capacity from the Ogallala Aquifer.

The environmental and economic advantages of cover crops have been widely reported [3,4,5]. Reducing soil erosion, increasing water infiltration and soil organic matter, and supplying nutrients to successive crops are the most relevant benefits for the SHP. Despite the well-recognized benefits of cover crops, their adoption in the SHP has been minimal. Erratic weather patterns and unpredictable market conditions make producers hesitant to plant crops that may not generate immediate economic benefits [4,6].

Perennial forage grasses dominate grazing systems in this semiarid region, specifically ‘WW-B. Dahl’ Old World bluestem [OWB, *Bothriochloa bladhii* (Retz). T. Blake] and native grasses such as buffalo grass [*Buchloe dactyloides* (Nutt.) Engelm], blue grama [*Bouteloua gracilis* (Willd. ex Kunth) Lag. ex Griffths], and sideoats grama [*Bouteloua curtipendula* (Michx.) Torr]. The most limiting nutrient in the weight gain of steers grazing in perennial pastures is often crude protein (CP) [7,8,9]. High-protein annual forages can complement perennial pastures to maintain productivity in the system [10]. For instance, summer annual forages such as teff [*Eragrostis tef* (Zucc.) Trotter] are gaining popularity among producers looking for fast-growing forages with low water and fertilizer requirements and higher nutritive value. Teff would pair well in an annual forage rotation with winter annual forages managed as a cover crop.

Unfortunately, there is a dogma among landholders in the SHP that cover crops extract too much water from the soil profile, hindering the productivity of the successive cash crop (unpublished research observation). Managing the vegetative cover as a residue that improves soil productivity for longer-term benefits, with added potential as a forage source, can help negate these concerns. This research investigates the interacting effects of irrigation and tillage with five cover crops on production, forage nutritive value, and soil water depletion of the cover crops and subsequent teff summer hay crop to identify low-risk cover crop practices in the drought-prone SHP.

## 2. Materials and Methods

### 2.1. Description of Research Site

This research was conducted in Lubbock, TX (33°45′ N, 101°47′ W; 993 m elevation) between September 2015 and September 2017. The site contained Pullman clay-loam soils with a thick layer of CaCO_3_ (caliche) at depths of 60 to 120 cm. The research site was nearly flat, with 0 to 1% slopes. The monthly rainfall ranged from 15 to 65 mm, and the potential evapotranspiration ranged from 90 to 290 mm. This variability resulted in very different production seasons in each year (Table 1). Plots were equipped with an underground drip irrigation system with a turbine water meter (Master Meter WNT-01, Fort Worth, TX, USA) to track the amount of irrigation applied. Irrigation was applied by hand, with a portable water tank, during the winter months when the drip lines were drained to prevent freezing. In Year 1, one access tube for a Dynamax PR2 Profile Probe System (Dynamax Inc., Houston, TX, USA) soil moisture (capacitance) sensor was installed in each sub-subplot in the central block (block 2 of 3; 24 total tubes).

### 2.2. Experimental Design and Plot Management

**Figure 1:**
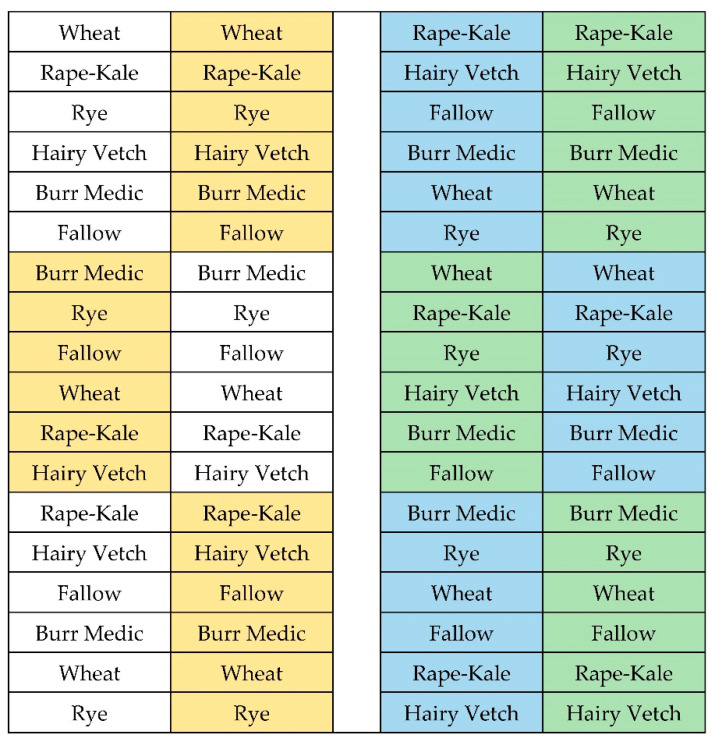
Orientation of treatments when winter cover crops were planted. Irrigation treatments were applied across all blocks (whole plot factor). Tillage treatments were randomly arranged east-west within whole plots (subplot factor). Cover crop treatments were applied perpendicular across subplots (sub-subplot factor). Color blocks represent treatment: white = dryland, no-till, yellow = dryland, minimum till, blue = irrigated, no-till, and green = irrigated, minimum till.

A 15 m × 55 m area was allocated to this strip-split-block design, small-plot experiment, with three replicate blocks (Figure 1). Two winter irrigation treatments, dryland and irrigated, were applied across all blocks as a strip (whole plot factor). Irrigation supplemented rainfall up to 25 mm per month of total water (rain + irrigation) delivery in the winter irrigation treatment. Irrigation was only applied when volumetric water content (VWC) measured at 10 cm fell below 15%. Each whole plot (block × irrigation combination) contained two tillage treatments (minimal and no-till, with subplot strips randomly arranged east-west within whole plots) perpendicular to six cover crop treatments (five species and one fallow control; subsub-plots were randomly arranged north-south across tillage x irrigation x block subplots). Minimum tillage consisted of lightly disking 5 cm deep with a tractor-mounted power-tiller to break the surface crust. All plots were minimally tilled in Year 1 to terminate established perennial grasses.

Forage treatments compared rye (cv. Maton II), wheat (*Triticum aestivum* L.; cv. TAM204), burr medic (*Medicago polymorpha* L.; cv. Armadillo), hairy vetch (*Vicia villosa* Roth; cv. Vallana), rape-kale (*Brassica* spp.; cv. Bayou), and an unplanted fallow. Legumes were inoculated with the appropriate *Rhizobium* bacteria prior to being planted each year. Cover crops were drilled into 3 m × 3 m plots on 18 cm row spacing using a Tye 2010 Stubble Drill (Tye Manufacturing) in mid-September of 2015 and 2016, at planting rates listed in Table 2. No fertilizer or pesticides were applied to the cover crops.

Teff (cv. Tiffany) was planted using the same drill each May to measure its response to cover crop treatments (Table 2). All teff (regardless of winter irrigation) was drip-irrigated to supplement rainfall up to 127 mm per month of total water delivery in May through August. The teff was fertilized with ammonium nitrate at 56 kg N ha^−1^ at planting and after the first harvest. No pesticides were applied to the teff.

### 2.3. Forage Responses

The extent of legume nodulation (1–5 visual scale) was recorded in five locations within each cover crop plot before harvest. The forage mass of the cover crops was determined each spring from three 0.3 m × 1 m quadrat samples hand-clipped to an 8-cm stubble height. A flail harvester with a 1-m cutting width was used to harvest a 2.5-m-long strip to 8-cm stubble height from each teff plot in Year 1. Plot weight was recorded before collecting a subsample to determine dry matter and nitrogen concentration. Because of mechanical problems and labor constraints in Year 2, teff forage mass was determined by hand clipping one 0.3-m^2^ sample from each plot. Residual biomass was removed from all harvested experimental areas to maintain vegetative growth.

### 2.4. Crude Protein

Forage subsamples were ground to pass a 1 mm sieve using a Wiley Mill (Thomas-Wiley Laboratory Mill, Thomas Scientific, Swedesboro, NJ, USA) before they were submitted to A & L Plains Laboratories in Lubbock for determination of plant N concentration by dry combustion using a LECO analyzer [11]. Crude protein concentration was calculated by multiplying plant N by 6.25. Cover crop samples were composited across blocks because of limited sample material.

### 2.5. Soil Water Responses

Soil volumetric water content (VWC) was monitored weekly (except at times of equipment malfunctions or inclement weather) with the Dynamax PR2 Soil Profile Probe at 10, 20, 30, 40, 60, and 100 cm depths. Despite the setbacks from equipment malfunctions and the weather, readings were still recorded at least once per month for the entire trial period.

### 2.6. Statistical Analyses

Normality was confirmed prior to any further statistical analysis with the Shapiro-Wilk test within Proc Univariate in SAS 9.4 (SAS Inst., Cary, NC, USA). Treatment comparisons were made by restricted maximum likelihood with mixed-model analysis of variance using Proc Mixed, with additional analyses done within the year where appropriate [12]. A Kenward–Rogers adjustment was applied to correct the denominator degrees of freedom, ensuring appropriate standard errors and F statistics for each model. The irrigation strip design across the blocks disallowed statistical testing of the main irrigation effect; however, all interactions with tillage and cover crop treatments were tested [13]. A full list of the main effects is provided in Table 3. Means were compared using the LSMEANS procedure, and the differences were considered significant at *p* < 0.05.

## 3. Results

### 3.1. Cover Crop Production

#### 3.1.1. Accumulated Forage

**Figure 2:**
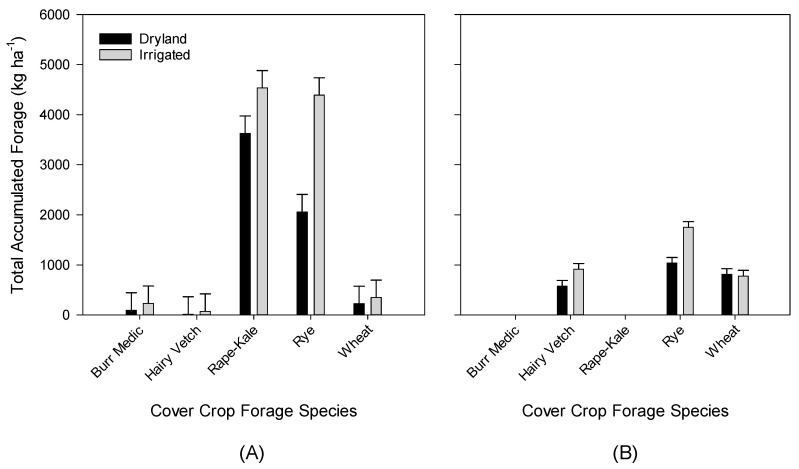
Total accumulated forage produced by each irrigation x cover crop forage species combination within each year. (**A**) Data were collected on 9 March, 23 March, and 22 April 2016 in Year 1 (*n* = 36). (**B**) Data were collected on 6 March and 28 April 2017 in Year 2 (*n* = 72). Data averaged over blocks and tillage treatment (applicable in year 2 only). Error bars represent standard error.

The total accumulated forage observed was greater in Year 1 than Year 2, so treatment differences were analyzed within each year (*p* < 0.01). The interaction of irrigation and forage treatment affected the total accumulated forage within both years (*p* < 0.02; Figure 2). All other interactions were not significant (*p* > 0.05).

In Year 1, dryland rape kale, irrigated rape kale, and irrigated rye produced the greatest total accumulated forage, followed by dryland rye (*p* = 0.02, Figure 2A). The remaining cover crops produced negligible forage mass (<400 kg ha^−1^). In Year 2, the greatest total accumulated forage was produced by irrigated rye (*p* < 0.01, Figure 2B). This was followed by dryland rye; however, the total accumulated forage was not greater than other irrigation x forage combinations, except for dryland hairy vetch (*p* = 0.04). The burr medic and rape kale stands did not produce a harvestable mass in Year 2.

#### 3.1.2. Crude Protein Concentration

**Figure 3:**
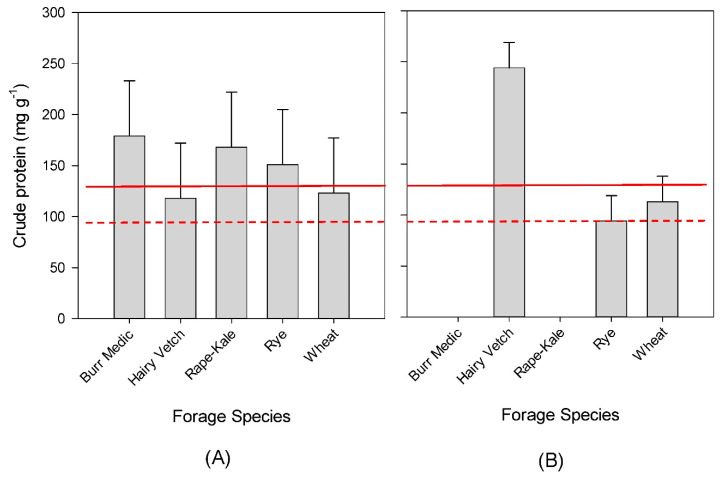
Comparison of crude protein concentration of cover crop forage species within each year. (**A**) Data were collected on 9 March, 23 March, and 22 April 2016 in Year 1 (*n* = 14). (**B**) Data were collected on 6 March and 28 April 2017 in Year 2 (*n* = 20). Data averaged over irrigation and tillage treatments (applicable in Year 2 only). Error bars represent standard error. The solid red line represents approximate protein requirements of growing steers, and the dashed red line represents the requirements of mature beef cattle.

Crude protein concentration was analyzed within each year because of crop failures in Year 2 (Figure 3). There were no differences in CP concentration in Year 1 (*p* = 0.57, Figure 3A); however, all forages would have met or exceeded the protein needs for mature or growing cattle. Crude protein concentration differed by forage species in Year 2 (*p* < 0.01, Figure 3B). Hairy vetch generated the greatest CP concentration, and there was no difference in wheat and rye. Hairy vetch exceeded the protein requirements for even growing steers, while the small grains may fall short.

#### 3.1.3. Nodulation

Forage species was the only factor identified that affected nodulation within each year. In both years, hairy vetch produced more active nodules than burr medic (*p* < 0.01). Nonetheless, evidence of nodulation was rare in both species. The average nodulation scores were 0.08 and 0.55 for burr medic and hairy vetch, respectively, in Year 1. Likewise, burr medic showed less evidence of nodulation in Year 2 (0.02), whereas hairy vetch increased (1.3).

### 3.2. Teff Production

#### 3.2.1. Accumulated Forage

**Figure 4:**
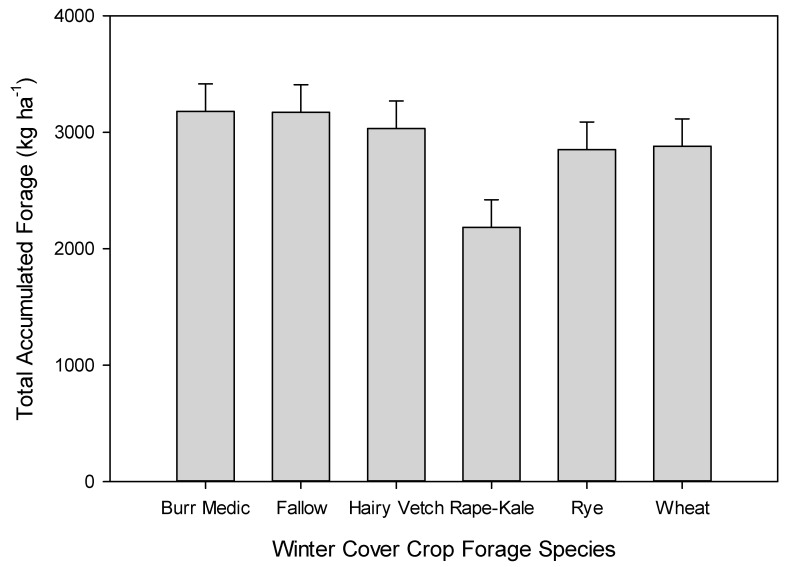
Total accumulated forage produced by teff following winter cover crops in Year 1. Data are averaged over blocks and irrigation treatments. Data were collected on 23 July and 2 September 2016 (*n* = 72). Error bars represent standard error.

In Year 1, competition from the winter-planted rape kale decreased the total accumulated forage of teff in comparison to the other cover crops (*p* < 0.01; Figure 4). All other interactions were not significant (*p* > 0.05). Numerically, the average total accumulated forage was much greater in Year 2 (3829 kg ha^−1^) compared to Year 1 (2884 kg ha^−1^). However, none of the main effects or their interactions affected the total accumulated teff production in Year 2 (*p* = 0.08).

#### 3.2.2. Crude Protein Concentration

Crude protein concentration was analyzed within each production year since weather delays prevented timely harvests in Year 2, which resulted in more mature forage that was lower in CP (162 mg g^−1^ and 98 mg g^−1^, respectively). The main effects and their interactions did not impact CP concentration in Year 1 of this study (*p* = 0.30). Winter forage species was the only significant effect in Year 2 (*p* = 0.02). Teff grown following hairy vetch resulted in the greatest numerical CP concentration (106 mg g^−1^); however, it was only statistically greater than teff grown after rye and wheat (95 mg g^−1^ and 88 mg g^−1^, respectively).

### 3.3. Impact on Soil Water Status

#### 3.3.1. Irrigation Applied

Less irrigation was applied during the winter cover crop growth period between September to April in Year 1 (54 mm) than in Year 2 (84 mm) (*p* < 0.01) to meet the target of 25 mm of water received by rain and irrigation per month when soil VWC at 10 cm depth fell below 15%. This was consistent with Year 1 having less rainfall (Table 1). All other main effects and their interactions were not significant (*p* > 0.05). Teff received 297 mm of irrigation in Year 1 and 229 mm in Year 2, reflecting the greater amount of irrigation needed to supplement the greater deficit of summer rainfall in Year 1.

#### 3.3.2. Volumetric Water Content

**Figure 5:**
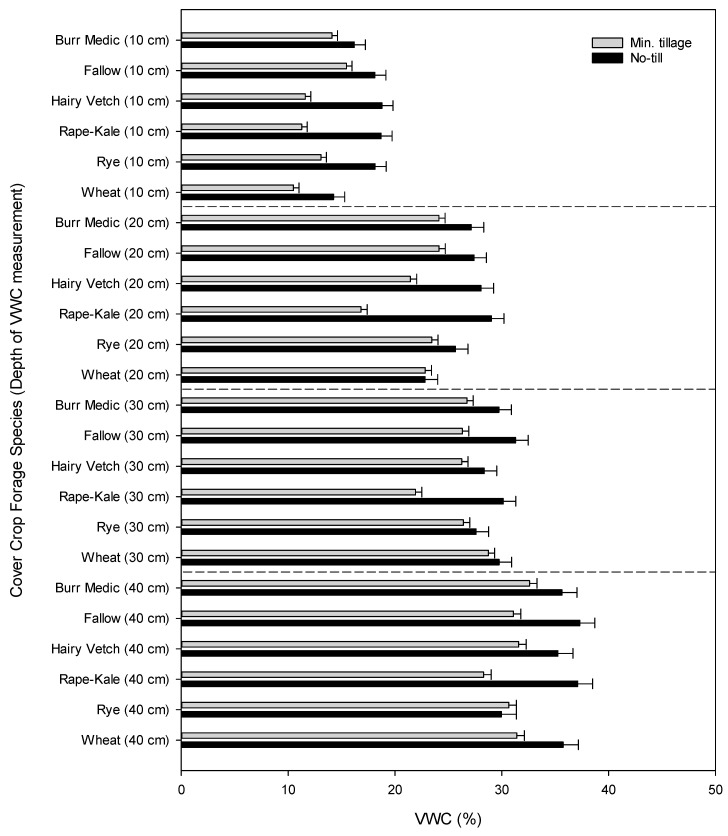
Comparison of average weekly volumetric water content at four depths: 10 cm, 20 cm, 30 cm, and 40 cm. Data were analyzed within depth and averaged over irrigation treatments. Fallow represents the unplanted control. Error bars represent standard error.

Volumetric water content was affected differently at each of the six tested depths. Tillage and forage species interacted to affect VWC at depths of 10, 20, 30, and 40 cm (*p* < 0.01; Figure 5). Volumetric water content was reduced in all forage treatments, except burr medic at 10 cm deep when tilled. Tillage reduced VWC in all forages except for rye and wheat at a depth of 20 cm. Hairy vetch, rye, and wheat were not affected by tillage at a depth of 40 cm. Tillage did not reduce VWC in burr medic and rye at 60 cm deep.

To simplify the discussion, we now focus on comparisons to the no-till, dryland, winter fallow treatment (control). Nearly all minimal tillage x forage species combinations reduced VWC at depths of 10, 20, 30, and 40 cm, compared to the no-till control (*p* < 0.05). No-till wheat resulted in a lower VWC at depths of 10 and 20 cm, while no-till rye reduced VWC at depths of 30 and 40 cm compared to the control (*p* < 0.05).

The interaction of irrigation and tillage only impacted VWC at a depth of 20 cm. This can most likely be attributed to the location of the underground drip tape. Tillage reduced VWC more in irrigated plots than dryland (*p* < 0.01, Table 4). Irrigation, tillage, and winter forage species interacted to affect VWC at depths of 60 and 100 cm (*p* < 0.01, data not shown). However, there are no logical trends in the results, and it is impossible to separate treatment differences from possible environmental, soil, or equipment effects.

## 4. Discussion

**Figure 6:**
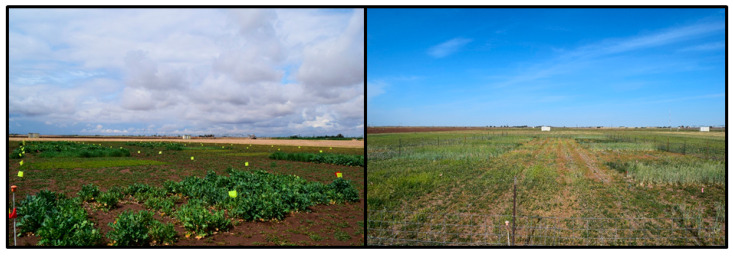
View of cover crop growing in the experimental plot area in Year 1 (spring 2016; **left**) and Year 2 (spring 2017; **right**). Crops grown included: rye (cv. Maton II), wheat (*Triticum aestivum* L.; cv. TAM204), burr medic (*Medicago polymorpha* L.; cv. Armadillo), hairy vetch (Vicia villosa Roth; cv. Vallana), rape kale (*Brassica* spp.; cv. Bayou), and an unplanted fallow.

The timing and volume of precipitation events were critical to the success of the crops each year. Even though burr medic produced a decent ground cover in Year 1 (visually estimated at 75%), the crop never accumulated significant mass that could be harvested (<300 kg ha^−1^). The chronic soil water deficits and high summer heat contributed to a challenging environment for *Rhizobium* survival and activity in West Texas soil. Despite having inoculated the seeds, poor nodulation may have contributed to low forage production of the legumes. Hairy vetch produced much greater total accumulated forage in Year 2, which may indicate a high level of dormant seed planted in Year 1. It was surprising that rape kale performed so well in Year 1 and then failed in Year 2. Although the seedlings emerged, they did not persist under the colder, drier weather that occurred in autumn in Year 2. Irrigated rye was consistently the highest producing crop in both years of the study, while dryland rye closely followed. The wheat crop produced measurable forage mass both years, but it never matched the irrigated rye. The wheat was suppressed in Year 1 by grazing from wildlife, and delayed regrowth after an extended period under snow (over 7 days). These differences between forage species are further illustrated in Figure 6, which illustrates the drier conditions and lower overall forage growth in Year 2 compared to Year 1.

Our results indicate that timely termination of the cover crop is critical in order to prevent yield loss in the summer cash crop. Delayed termination of the cover crop was the most significant factor that reduced the productivity of the summer teff crop. In Year 1, strong northerly winds prevented chemical termination of the cover crops (cotton was planted directly south of experiment), and close mowing did not sufficiently terminate the rape kale, which continued to grow throughout early summer, to the detriment of the summer teff crop. This supports Robinson and Neilsen’s argument that the most valuable winter cover available to a producer is the residue from the previous summer’s cash crop because of the risk of greater stress on the next summer’s crop following the winter cover crop [14].

However, there are several reports of cover crops used succesfully in rotation with cash crops. Recently, an experiment in semi-arid eastern New Mexico (U.S.A.) reported no impact of winter cover crops on productivity in a corn-sorghum (*Zea mays, Sorghum bicolor*) rotation [15]. This research was conducted with pea (*Pisum sativum* L.), oat (*Avena sativa*), canola (*Brassica napus*), and their combinations. The authors recommended no-till management with careful consideration of planting and termination dates to ensure production of the cash crop [15]. Similarly, cover crops did not reduce cotton (*Gossypium hirsutum*) yields or net returns when managed under dryland conditions in the Texas Rolling Plains [16]. This research was conducted on winter pea, hairy vetch, crimson clover (*Trifolium incarnatum* L.), wheat, and multi-species mixtures. There is a surprising paucity of information about growing cover crops in the Southern High Plains, especially for grazing purposes.

Crude protein is usually the most limiting nutrient in grazing beef cattle diets in the SHP [9]. Protein supplementation is often required for the warm-season perennial forages that dominate in the region, since the average CP usually falls below the 80 mg g^−1^ required for mature, non-lactating beef cows. Winter annual forages are relatively high in digestible energy and protein concentrations, especially when compared to dormant grasses or hay.

Winter cover crops, particularly small grains, provide many advantages to integrated crop-livestock systems, but management can modulate the benefits [17]. These authors also note the importance of planting date, grazing intensity, and weather when trying to maximize the benefits of the cover crop. Winter wheat dominates much of the Great Plains for cool-season forage [17]; however, wheat did not reliably produce as much forage as rye in the current study.

It is not surprising that hairy vetch had a greater CP concentration in Year 2 of this experiment. It is well known that legumes tend to have higher CP than grasses. Unfortunately, the legumes tested did not reliably generate enough forage to sustain grazing livestock. Future research endeavors should evaluate other legume species or hairy vetch in combination with small grains to find an optimum balance between forage mass and nutritive value.

There was not a clear trend in irrigation required by the different crops, probably because the variable successes of crops in stand establishment between the years drove water use differences more than inherent crop water requirements. The impact of winter cover crop management on soil VWC across the season was notable. Based on the results presented in Figure 5, it is important for producers to no-till plant cover crops, since even light tillage decreased VWC down to a depth of 40 cm. No-till planted crops generally did not reduce soil VWC compared to the unplanted fallow in this depth range, except for the small grains. No-till wheat likely reduced the VWC in the top 20 cm of the soil because of its limited ground cover and shallow root system (unpublished research observation). The no-till rye did reduce VWC at 30 and 40 cm below the soil surface compared to the unplanted fallow, but it was not to the detriment of the summer cash crop. One possible explanation is that teff is known to be shallow rooted; thus, teff productivity was less vulnerable to the depressed VWC at the lower depths [18].

## 5. Conclusions

The results of this research should help negate concerns from producers in the SHP who avoid planting winter cover crops because of predicted detrimental impacts on the summer cash crop, as indicated by our use of teff as an indicator cash crop. Rye produced the greatest accumulated forage and was the most reliable forage tested. Even in a dry year, dryland rye produced modest forage biomass. However, light irrigation should be used to ensure adequate forage supply if the producer is reliant upon the crop for winter grazing. No-till management and timely termination of the winter cover crop were crucial to reduce the impact of winter crops on summer teff production. The results indicate that there was no detriment to soil water content that was attributable to planting no-till cover crops compared with the conventional practice of winter fallow. Therefore, producers could take advantage of the soil-conserving attributes of high-quality winter forage cover crops without significant soil water depletion. Future research objectives should include identifying the optimum termination date for the winter crop, calculating the economic advantages of cover crops managed under multiple irrigation and tillage strategies, and exploring combinations of winter cover crops.

## Figures and Tables

**Table 1:** Seasonal rainfall total (mm) for Lubbock, TX. Long-term mean is 106-year average from the nearest National Oceanic and Atmospheric Administration (NOAA) station (Lubbock airport).

Season	Year 1	Year 2	Long-Term Mean
September to April	218	178	236
May to August	236	353	234
Total September to August	455	531	470

**Table 2:** Summary of seeding rates for all forage species.

Forage Species	Seeding Rate, kg Pure Live Seed ha^−1^
Burr Medic (*Medicago polymorpha* L.; cv. Armadillo)	22
Hairy Vetch (*Vicia villosa* Roth; cv. Vallana)	17
Rape-Kale (*Brassica* spp.; cv. Bayou)	11
Rye (*Secale cereale*; cv. Maton II)	67
Wheat (*Triticum aestivum* L.; cv. TAM204)	67
Teff (*Eragrostis tef* (Zucc.) Trotter; cv. Tiffany)	10

**Table 3:** Summary of main effects tested for each statistical model.

Model	Fixed Effects	Random Effects
Total season accumulated forage (cover crops)	tillage, forage, irrigation × tillage, irrigation × forage, tillage × forage, irrigation × tillage × forage [analyzed within year]	block, block × irrigation, block × tillage, block × forage
Total season accumulated forage (teff)		
Crude protein concentration (teff)		
Nodulation (cover crops)		harvest
Crude protein concentration (cover crops)		
Volumetric water content	tillage, forage, irrigation × tillage, irrigation × forage, tillage × forage, irrigation × tillage × forage	week
Irrigation applied (cover crops)	tillage, forage, year, tillage × forage	none

**Table 4:** Differences in average weekly volumetric water content measured at 20 cm soil depth under different winter irrigation regimes.

Winter Irrigation Treatment	Average Weekly Volumetric Water Content, %	
	No-Till	Minimum Tillage
Dryland	23.9	21.5
Irrigated	29.5	22.8

## Data Availability

The data presented in this study are available on request from the corresponding author.
